# Parametric Study of Amorphous High-Entropy Alloys formation from two New Perspectives: Atomic Radius Modification and Crystalline Structure of Alloying Elements

**DOI:** 10.1038/srep39917

**Published:** 2017-01-04

**Authors:** Q. Hu, S. Guo, J.M. Wang, Y.H. Yan, S.S. Chen, D.P. Lu, K.M. Liu, J.Z. Zou, X.R. Zeng

**Affiliations:** 1 Shenzhen Key Laboratory of Special Functional Materials, College of Materials Science and Engineering, and Key Laboratory of Optoelectronic Devices and Systems of Ministry of Education and Guangdong Province, College of Optoelectronic Engineering, Shenzhen University, Shenzhen 518060, China; 2 Surface and Microstructure Engineering Group, Materials and Manufacturing Technology, Chalmers University of Technology, SE-41296, Gothenburg, Sweden; 3 Institute of Applied Physics, Jiangxi Academy of Sciences, Nanchang, 330029, China

## Abstract

Chemical and topological parameters have been widely used for predicting the phase selection in high-entropy alloys (HEAs). Nevertheless, previous studies could be faulted due to the small number of available data points, the negligence of kinetic effects, and the insensitivity to small compositional changes. Here in this work, 92 TiZrHfM, TiZrHfMM, TiZrHfMMM (M = Fe, Cr, V, Nb, Al, Ag, Cu, Ni) HEAs were prepared by melt spinning, to build a reliable and sufficiently large material database to inspect the robustness of previously established parameters. Modification of atomic radii by considering the change of local electronic environment in alloys, was critically found out to be superior in distinguishing the formation of amorphous and crystalline alloys, when compared to using atomic radii of pure elements in topological parameters. Moreover, crystal structures of alloying element were found to play an important role in the amorphous phase formation, which was then attributed to how alloying hexagonal-close-packed elements and face-centered-cubic or body-centered-cubic elements can affect the mixing enthalpy. Findings from this work not only provide parametric studies for HEAs with new and important perspectives, but also reveal possibly a hidden connection among some important concepts in various fields.

High entropy alloys (HEAs) are multi-component alloys with four or more elements mixed in equiatomic or close-to-equiatomic ratios^1^,^2^,^3^,^4^. The name of “high entropy” comes from the high entropy of mixing (Δ*S*_mix_, 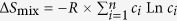, where *R* is the gas constant, *n* is the number of alloying elements, *c*_i_ is the atomic percentage for the *i*th element) of an alloy. Δ*S*_mix_ reaches the maximum when its constituent elements have equiatomic ratios, i.e., 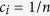. Interestingly, even with many elements mixed with a high concentration, simple phases such as solid solution^5^,^6^,^7^,^8^,^9^ and sometimes metallic glasses (MGs, or amorphous alloys)^10^,^11^,^12^,^13^,^14^,^15^,^16^ tend to form in a large number of HEAs, without the formation of complex intermetallic compounds. Solid solution forming alloys have been developed in conventional alloys where there exits only one dominant element, and amorphous alloys used to be explored near eutectic compositions^17^,^18^. With the design concept of HEAs becoming more accepted by the materials community, more and more simple face-center cubic (FCC), body-center cubic (BCC), and hexagonal close-packed (HCP) solid solution forming HEAs and amorphous HEAs have been developed, some of which exhibit good mechanical^19^,^20^,^21^,^22^,^23^, physical^24^,^25^,^26^,^27^, chemical^28^ and biomedical^13^ properties. Therefore, HEAs provide new opportunities to design new alloys with potentially new properties. The alloy design for HEAs is a complex issue, considering their compositional complexity and the possibility of forming various phases in them, including solid solutions (mainly of FCC, BCC and HCP structure), intermetallic compounds and the amorphous phase. Therefore, phase selection is the first and foremost step for the alloy design of HEAs. This step is especially crucial for designing amorphous alloys in HEAs, since there is little information available that can be used. For example, amorphous alloys have been explored near eutectic alloys in conventional alloys. However, due to the lack of well-established thermodynamic databases for HEAs, locating eutectic compositions in HEAs is not straightforward^29^,^30^. On the other hand, even in known eutectic HEAs, rapid solidification did not lead to the formation of amorphous alloys^29^.

So far, the alloy design for HEAs has relied more on empirical methods, utilizing the parametric approach^5^,^31^,^32^,^33^,^34^,^35^,^36^,^37^,^38^. Indeed, the parametric approach has been proven to be very useful in guiding the phase selection. Two types of parameters, one out of chemistry nature and the other one out of topology nature, are generally employed in combination in parametric approaches. The mixing enthalpy, Δ*H*_mix,_ is the most widely used chemical parameter^39^,^40^,^41^,


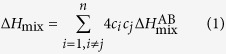


where 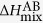 is the mixing enthalpy of equiatomic binary liquid AB alloys. The most widely used topological parameter is the atomic size polydispersity^33^,^42^,^43^, *δ*,


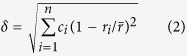


where 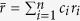, *r*_*i*_ is the atomic radius of the *i*th element. A different topological parameter, *γ*, emphasizes the effect of the largest and smallest atoms^36^,


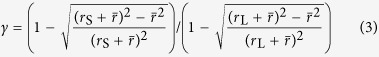


where *r*_S_ and *r*_L_ are atomic radii of the smallest and largest atoms. Another parameter, *α*_2_, aims to describe the lattice distortion using the dimensionless displacement between an atomic pair and its counterpart pair^37^,


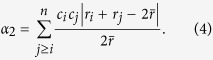


Last but not least, there is the parameter, <*ε*^2^>^1/2^, which is the root-mean-square residual strain^44^


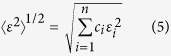


where


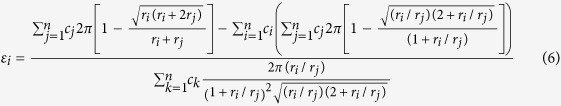


Although different expressions are used and the thinking behind them are also different, the above mentioned four topological parameters, *δ, γ, α*_2_ and <*ε*^2^>^1/2^, all try to quantify the atomic size mismatch of an alloy.

Reviewing of existing parametric approaches seems to point to one regularity on the phase selection in HEAs, i.e., solid solutions form when indices for chemical parameters are large and those for topological parameters are small (for example, Δ*H*_mix_ is not slightly negative or even position, and *δ* is small); by contrast, amorphous alloys form when indices for chemical parameters are small and those for topological parameters are large (for example, Δ*H*_mix_ is highly negative, and *δ* is large); intermetallic compounds normally form in intermediate conditions in terms of the index for chemical and topological parameters. This regularity, however, needs to be further inspected due to the following considerations.

Firstly, HEAs investigated before in general have a wide distribution of alloy compositions, which could widen the gap of parameters and result in a clear separation in parameters for different phases. An alloy system with a narrower compositional distribution is therefore more appropriate, as the parameters would be less compositional sensitive and it requires the really useful parameters to be genuinely phase sensitive^45^,^46^. It is intriguing to know whether the above mentioned regularity can still stand in such an alloy system. Secondly, the number of equiatomic MGs is too small, when compared to the thousands of traditional MGs based on one or two principal elements. More equiatomic MGs are thus required to enlarge the database to verify the regularity. Thirdly, the kinetic effect on the phase selection, especially for the amorphous phase, needs to be completely eliminated, since it is well known that kinetic factors like the cooling rate in solidification has a great effect on the formation of the amorphous phase^34^,^47^. Alloys prepared under the same condition, for example, all by rapid quenching, are more suitable as target materials to verify the phase selection rules. Fourthly, in the above mentioned four topological parameters (also in all reported topological parameters), the atomic radius of pure elements^48^, rather than the atomic radius of these elements in specific alloys (specific atomic environment) are used. The atomic radius is usually defined as the half distance between an atom and its nearest one. Therefore, for any element, its atomic radius in pure metals and in alloys are different, due to the change in the local atomic environment. There is naturally a problem of using the atomic radius of a pure element to calculate the topological parameter of an alloy, although this problem did not stand out when dealing with alloys with a wide distribution of compositions.

Bearing these considerations in mind, 100 melt-spun equiatomic HEAs, all containing three same elements (Ti, Zr, Hf), are investigated in this work. About half of them are amorphous and thus provide a sufficient material database for checking the parametric approaches. The compositions in these 100 HEAs have a much narrower distribution compared to previously used material databases. It will be shown later that in such a specially designed alloy system with a narrow compositional distribution, none of the four topological parameters work in distinguishing the amorphous phase from crystalline ones (including solid solutions and intermetallic compounds, mainly intermetallic compounds). A simple but effective modification of the definition of the atomic radius, is proved to be able to solve the problem to a great extent. In addition, the significant effect of crystal structures of alloying elements on the mixing enthalpy is revealed here, for the first time.

## Results

**Figure 1:**
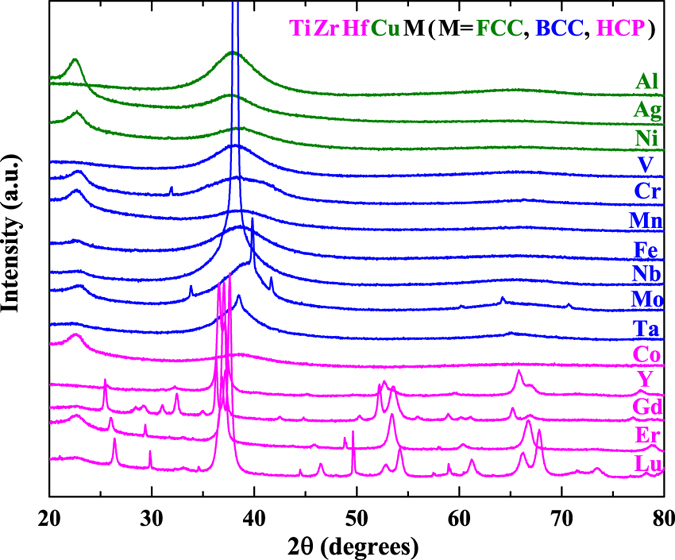
XRD patterns of TiZrHfCuM alloys.

In equiatomic HE-MGs, TiZrHfCuM (M = Fe, Co, Ni)^10^ are the first reported ones and TiZrHfCuNiBe^14^ has the largest dimension, indicating alloys based on TiZrHfCu can be potential candidates to investigate the amorphous phase formation in equiatomic HEAs. Fig. 1 shows the XRD pattern of TiZrHfCuM alloys, where M represents 15 typical elements, most of which are transition metals and rare earth metals. The results are classified by the crystal structure of the fifth component element, M. When M is a FCC metal (Al, Ag, Ni), a fully amorphous phase is formed in all cases; when M is a BCC metal (V, Cr, Mn, Fe, Nb, Mo, Ta), both amorphous and crystalline phase, and also their mixture are formed; when M is a HCP metal (Co, Y, Gd, Er, Lu), in most cases fully crystalline alloys are formed. Considering in this TiHfZrCuM alloy system, the first three constituent elements (Ti, Zr, Hf) are HCP metals and the fourth one (Cu) is a FCC metal, it seems that a combination of HCP and FCC metals favors the formation of the amorphous phase.

**Figure 2:**
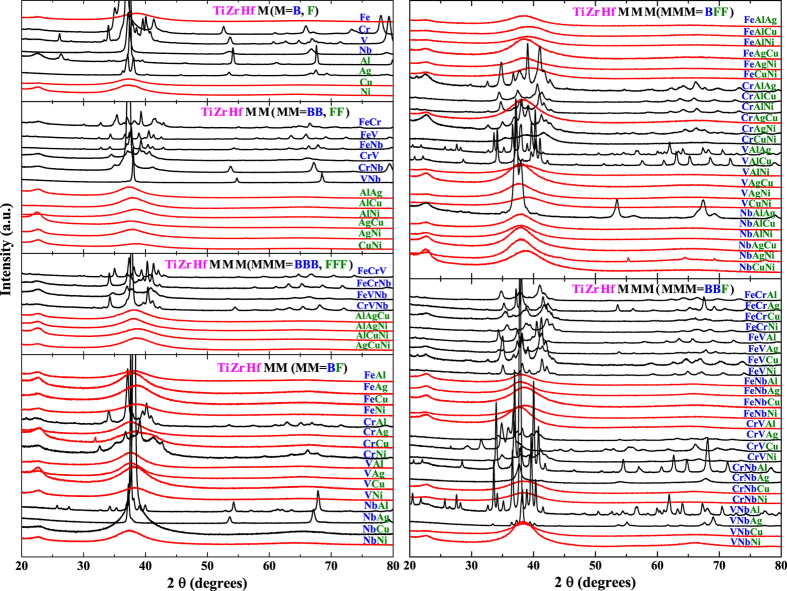
XRD patterns of TiZrHfM, TiZrHfMM and TiZrHfMMM alloys, where M represents FCC (Al, Ag, Cu, Ni) metal or BCC (V, Cr, Nb, Fe) metals. XRD patterns for crystalline and amorphous alloys, or otherwise regarded as amorphous alloys (refer to the full-text for clarification), are colored in black and red, respectively.

To further verify the above assumption, 4 FCC (Al, Ag, Cu, Ni) and 4 BCC (V, Cr, Nb, Fe) metals are employed to combine 3 fixed HCP metals (Ti, Zr, Hf), to form a sufficient large material database. All combinations of quaternary, quinary and senary alloys are investigated, including 8 (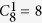) TiZrHfM (M = B, F), 28 (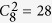) TiZrHfMM (MM = BB, FF, BF) and 56 (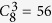) TiZrHfMMM (MMM = BBB, FFF, BFF, BBF). Here B and F are short for BCC and FCC metals, respectively. The XRD patterns of all 92 (8 + 28 + 56 = 92) alloys are shown in Fig. 2. Out of these 92 alloys, 43 form crystalline alloys and particularly 40 form intermetallic compounds, 47 form fully amorphous alloys, and 2 (TiZrHfCrCu, TiZrHfNbAgNi) form almost fully amorphous alloys but with a tiny amount of crystalline phases. For simplification, in the statistical analysis these 92 alloys are classified into two types of alloys: crystalline alloys, regardless solid solutions or intermetallic compounds, and amorphous alloys, regardless fully or almost fully amorphous.

**Figure 3:**
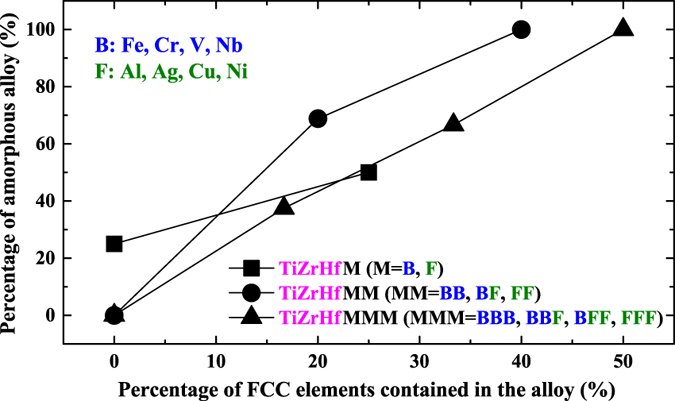
Relationship between the atomic ratio of FCC metal contents in TiZrHf (M, MM, MMM) alloys and the number ratio of achieved amorphous alloys among all alloys.

Clearly, almost all TiZrHf(F, FF, FFF) alloys are amorphous, and almost all TiZrHf(B, BB, BBB) are crystalline. A quantitative analysis showing how the portion of FCC metals in TiZrHf(M, MM, MMM) alloys can affect the amorphous phase formation is given in Fig. 3. Seen from Fig. 3, in all quaternary, quinary and senary alloys, a higher portion of FCC metals in the constituent elements means a larger chance for the amorphous phase formation. This statistical result lends support to our initial assumption. Figure 3 also indicates that a combination of HCP and BCC metals does not favor the amorphous phase formation.

**Figure 4:**
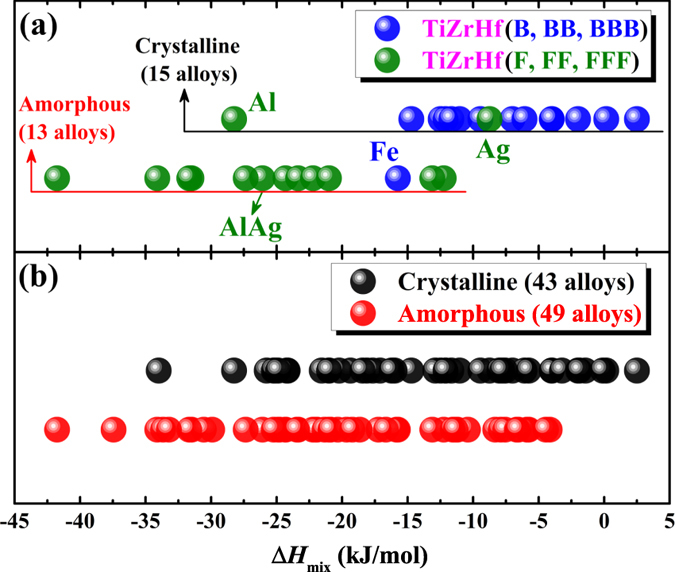
Chemical parameters for **(a)** crystalline and amorphous alloys containing only two types of elements, and **(b)** all crystalline and amorphous alloys.

The different effect of FCC and BCC elements on the amorphous phase formation is manifested most clearly in 28 alloys containing only two types of elements, i.e. TiZrHf(F, FF, FFF) and TiZrHf(B, BB, BBB), so only HCP and FCC, and only HCP and BCC elements. As shown in Fig. 4(a), Δ*H*_mix_ of most TiZrHf(F, FF, FFF) are more negative than those of TiZrHf(B, BB, BBB), indicating a more negative mixing enthalpy is in favor of the amorphous phase formation, which agrees with the regularity that is supported by parametric approaches^31^,^32^,^33^,^34^,^35^,^36^,^37^,^38^. In the meanwhile, Δ*H*_mix_ can also explain some exceptions on how the combination of HCP and FCC elements, or the combination of HCP and BCC elements can affect the amorphous phase formation. For example, TiZrHfFe has the most negative Δ*H*_mix_, −15.75 kJ/mol, which could explain why it is the only amorphous alloy in TiZrHfB series. Similarly, TiZrHfAg has the least negative Δ*H*_mix_, −8.75 kJ/mol, which renders it form the crystalline phase even it is in the TiZrHfF series. Therefore, Δ*H*_mix_ is vitally important (and apparently more important than the crystal structure of constituent elements) in making the difference on the amorphous phase formation, when combining FCC or BCC metals with HCP metals. In addition, Fig. 4(b) shows that for a total of 92 alloys, Δ*H*_mix_ also has some effect in distinguishing the amorphous phases from the crystalline ones.

**Figure 5:**
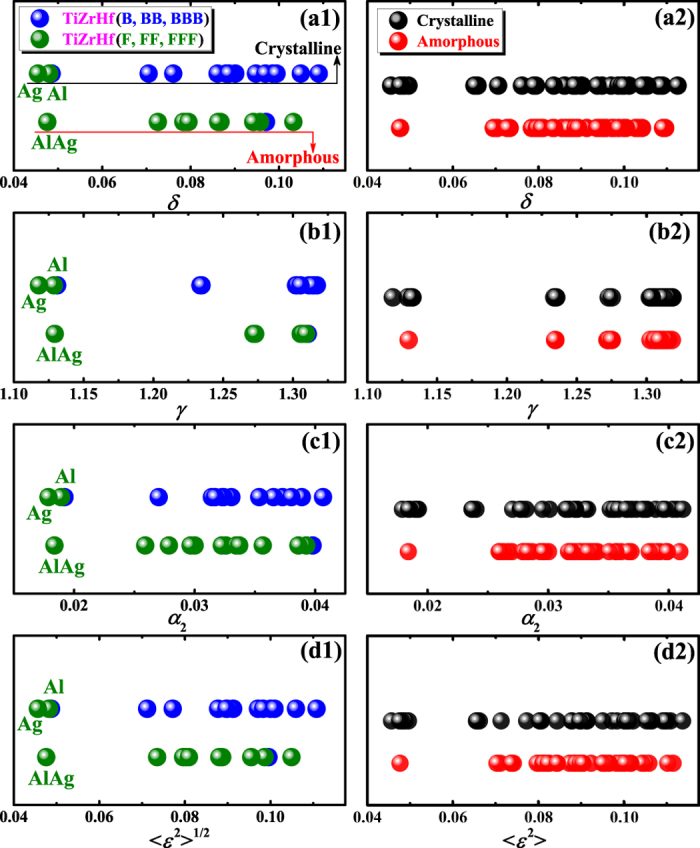
Topological parameters for **(a1–d1)** crystalline and amorphous alloys containing only two types of elements, and **(a2–d2)** all crystalline and amorphous alloys.

It is now known that in addition to chemical parameters, topological parameters are also important for the phase selection in HEAs. Unfortunately, there is little difference among the four topological parameters in separating the formation of amorphous and crystalline alloys in TiZrHf(F, FF, FFF) and TiZrHf(B, BB, BBB) alloys, as shown in Fig. 5(a1–d1), and also in all amorphous and crystalline alloys, as shown in Fig. 5(a2–d2). All four topological parameters failed in distinguishing the formation of the amorphous phase from crystalline phases, even they have quite different definitions and each of them reflects the atomic size mismatch from a unique perspective. Considering in all four topological parameters, atomic radii for pure elements are used and the previously mentioned problem of using these atomic radii, it seems timely now to discuss how alternative definitions of atomic radii can help to solve the problem, in the current material database where the distribution of alloy compositions is intentionally designed to be narrow.

## Discussions

As argued above, using atomic radii from pure elements for topological parameters of alloys is problematic. Modified atomic radii, with justifiable physical meaning, need to be used for topological parameters to work effectively in distinguishing the formation of the amorphous phase from crystalline phases. Hard sphere assumption, in which the atom is assumed as an uncharged sphere with a fixed radius, is usually used in derivations of topological parameters due to the simplification reason. However, the atomic radius in reality is mostly determined by the electronic property, especially the electronic interaction between the nuclear charge(s) and outermost electron(s). Therefore, in this work atomic radii are modified by considering the electronic factors, to reflect the effect of local electronic environment on actual atomic radii in alloys.

**Figure 6:**
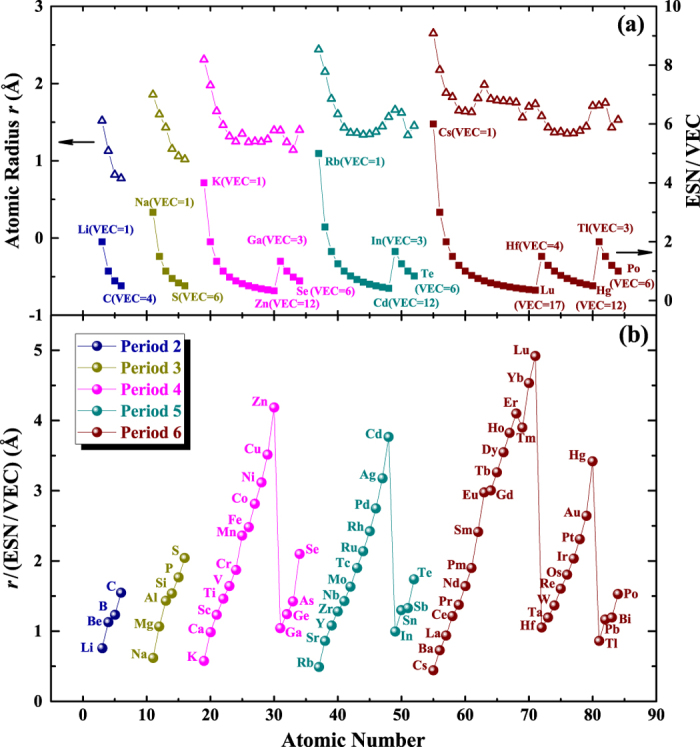
(**a**) Atomic radii and ESN/VEC, and **(b)** their ratio of the condensed-state elements.

Due to the shell structure of atoms, the atomic radius mainly depends on the electronic shell number (ESN) and the outermost electron number or valence electrons (i.e., valence electron concentration, VEC). ESN is also the period number, and VEC is also the group number for the elements of Group IA-IIB, and the group number minus 10 for elements of Group IIIA-VIIIA, except the lanthanides elements from La (VEC = 3) to Lu (VEC = 17)^49^. An extra shell usually means a larger space for the orbital motion of outer electrons. Therefore, in the same group, the atomic radius usually increases with increasing ESN, such as Ti and Zr, V and Nb, Cu and Ag, as listed in Table 1. Some exceptions do exist, particularly, the transition metals in Periods 5 and 6 have similar radii, so not sensitive to ESN, which is due to the complex effect of the electronic shell structure on the radius. For atoms with the same ESN, a larger VEC brings about opposite effects. One is that more outermost electrons would lie in a larger orbit, which would increase the radius. The other is that a larger VEC also means more protons and thus a higher nuclear charge, which brings stronger binding force to restrain the outermost electrons in a smaller orbit, i.e., the radius would decrease. In most cases, the atomic radius finally decreases because the latter effect usually prevails over the former. Considering the opposite contribution of ESN and VEC, ESN/VEC is proposed to represent the characteristics of the electronic shell structure of an atom, which is correlated to the atomic radius. As shown in Fig. 6(a), for all condensed-state elements, ESN/VEC and *r* show a similar trend and thus have a natural connection. However, due to the complexity of the electronic shell structure, its effect on the atomic radius is not the same for different elements, which can be seen from the different values of *r*/(ESN/VEC), as shown in Fig. 6(b). Actually, the ratio *r*/(ESN/VEC) has a clear periodic trend, indicating it is also an elemental characteristics that can represent the connection between the atomic radius and electronic shell structure.

In a pure metal, each atom has the same ESN and VEC and thus the same *r*. In an alloy, there are multiple elements with different ESN and VEC. Approximately, an element in an alloy can be assumed to have an average electronic shell number 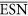 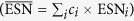 and an average number of valence electrons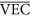 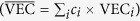. For the *i*th component element, supposing the connection between the atomic radius and electronic shell structure does not change much in pure metals and in alloys, i.e.,


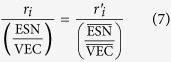


The modified atomic radius of the *i*th component element, when considering the change in local electronic environment in alloys, 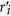, is thus defined as


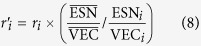


Here, 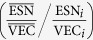 acts as a scaling factor introduced to reflect the effect of local electronic environment change on the atomic radius upon alloying.

Before employing the modified atomic radius to calculate topological parameters, its effectiveness needs to be verified. For pure metals, the atomic radius *r* is determined by the lattice parameter *a*, which can be precisely measured using XRD. For example, in BCC crystals, 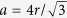. For an alloy, its lattice parameter *a*_alloy_ can be estimated by the well-known Vegard’s law^50^, i.e. 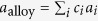, where *c*_*i*_ and *a*_*i*_ are the concentration and lattice parameter of the *i*th constituent element, respectively. Therefore, for alloys, the lattice parameter can be estimated using the atomic radii of the constituent elements. For example, for BCC alloys,


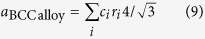


The Vegard’s law is an empirical rule and there usually have a deviation between the predicted and experimental values. An important reason for the deviation is believed to be the ignorance of the difference in the electronic structure of constituent elements, and some remediation considering the electronic factors are proposed to reduce the deviation^51^,^52^,^53^. Since the local electronic environment change upon alloying is indeed considered in the modified atomic radius in Eq. (8), it is believed that a better lattice parameter can be given by the following formula,


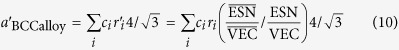


If this is indeed the case, the effectiveness of the modified atomic radii could be verified.

All 39 BCC alloys, constituted with the elements in Table 1 and are available in the powder diffraction file (PDF) database of the version PDF-2 Release 2004, are listed in Table S1 in Supplementary Information. As indicated by the signs in Table S1, in terms of the agreement between the experimental values and the values estimated from the Vegard’s law, *a*′ are better than *a* in 24 alloys and in most case *a*′ have an obviously advantage. *a*′ and *a* have the same or similar values in 12 alloys, where in 4 of them the modified radii are the same with the original ones, in 7 alloys both *a*′ and *a* have very good predictions with a deviation of less than 5‰, and in 1 alloy both *a*′ and *a* have very bad predictions with a deviation larger than 70‰. *a*′ is worse than *a* only in 3 alloys, especially for TiCr, which may be due to the imprecise *a*_exp_, since the quality of XRD pattern is labeled as poor in the PDF card of 65–9021. In brief, now it seems safe to say that the modified atomic radii can indeed reduce the deviation when using Vegard’s law to calculate the lattice parameter for alloys, at least in BCC alloys. In other words, the effectiveness of modified atomic radii can be verified.

The modified atomic radii of 92 HEAs are listed in Table S2 in Supplementary Information. According to Eq. (8), (ESN/VEC) has a great effect on the modified radii. As listed in Table 1, (ESN/VEC) are much smaller for Ag, Cu and Ni, which result in their much larger modified radii (see Table S2). On the other hand, (ESN/VEC) of Ti, Zr and Hf are greater than or equal to 1, which result in their modified radii being smaller than or equal to the original values (see Table S2). Therefore, the alloys containing these elements have large mismatch in the modified radii, which favors the amorphous phase formation. As shown in Fig. (2), in 49 amorphous alloys, 44 of them contain one or two or even three elements of Ag, Cu and Ni, which supports the above analysis. In addition, noting that Ag, Cu and Ni are FCC metals, the above analysis can thus also help explaining the regularity shown in Fig. (3), i.e., a combination of TiZrHf and FCC metals favors the formation of the amorphous phase. Furthermore, it seems not occasional that the above mentioned three FCC metals have smaller values of (ESN/VEC). Actually all FCC transition metals are located in Group VIII and IB with a large VEC of 9–11 and thus have a small (ESN/VEC) of 0.364–0.667. On the contrary, except Fe and Mn, all BCC transition metals are located in Group VB and VIB with a small VEC of 5 and 6, and thus have a large (ESN/VEC) of 0.667–1.200. The effect of electric shell structure on crystal structure of the transition metals have been well documented^54^. Now, in addition to pure metals, it is found that the electronic shell structure of the transition metals has also an effect on the phase selection in HEAs, when considering from the perspective of modified radii.

**Figure 7:**
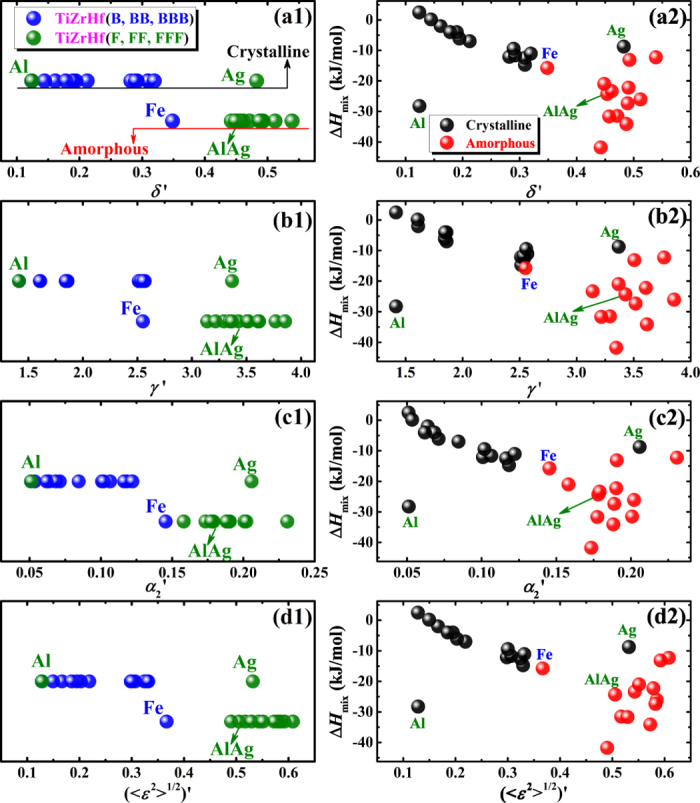
(**a1–d1**) Modified topological parameters and **(a2–d2)** Δ*H*_mix_ vs. modified topological parameters for crystalline and amorphous alloys containing only two types of elements.

Modified topological parameters, 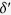,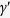, 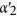 and 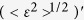 are calculated by substituting *r*_*i*_ in Eqs (2)–(6), 18, 20, 24, 10 by the modified atomic radius, 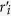. (see Supplementary Information Table S3). As shown in Fig. 7(a1–d1), the difference between amorphous alloys and crystalline alloys is now made more distinctive by modified parameters, compared to using original parameters that are shown in Fig. 5(a1–d1). Parameters for amorphous alloys are larger than those for crystalline alloys, indicating a large atomic size mismatch facilitates the amorphous phase formation, which again agrees with the regularity that is supported by parametric approaches^5^,^31^,^32^,^33^,^34^,^35^,^36^,^37^,^38^. The role of FCC and BCC metals played in the phase selection also becomes clearer. In most cases, TiZrHf(F, FF, FFF) alloys have larger topological parameters and more negative chemical parameters than those of TiZrHf(B, BB, BBB) alloys, and exceptions can be explained. For example, TiZrHfFe has the largest topological parameter, as shown in Fig. 7(a1–d1), and the most negative chemical value, as shown in Fig. 4(a), which makes it the only amorphous alloy in TiZrHf(B, BB, BBB) series. Although the topological parameters for TiZrHfAg get much larger after modification as shown in Figs 5(a1–d1) and 7(a1–d1), it still leads to crystalline alloys due to its unfavorable Δ*H*_mix_ of −8.8 kJ/mol, which is the least negative in TiZrHf(F, FF, FFF) series, as shown in Fig. 4(a). The significant improvement made by modification of atomic radii displays most obviously in crystalline TiZrHfAl and amorphous TiZrHfAlAg. They have close Δ*H*_mix_ of −28.3 and −24.3 kJ/mol respectively as shown in Fig. 4(a), and their original topological parameters are also close as shown in Fig. 5(a1–d1), which could not justify the formation of the amorphous phase and crystalline phases in them. After modification, the topological parameters for TiZrHfAl remain small, but those for TiZrHfAlAg increase sharply, can thus explain why the amorphous phase is formed in the latter alloy and not in the former.

**Figure 8:**
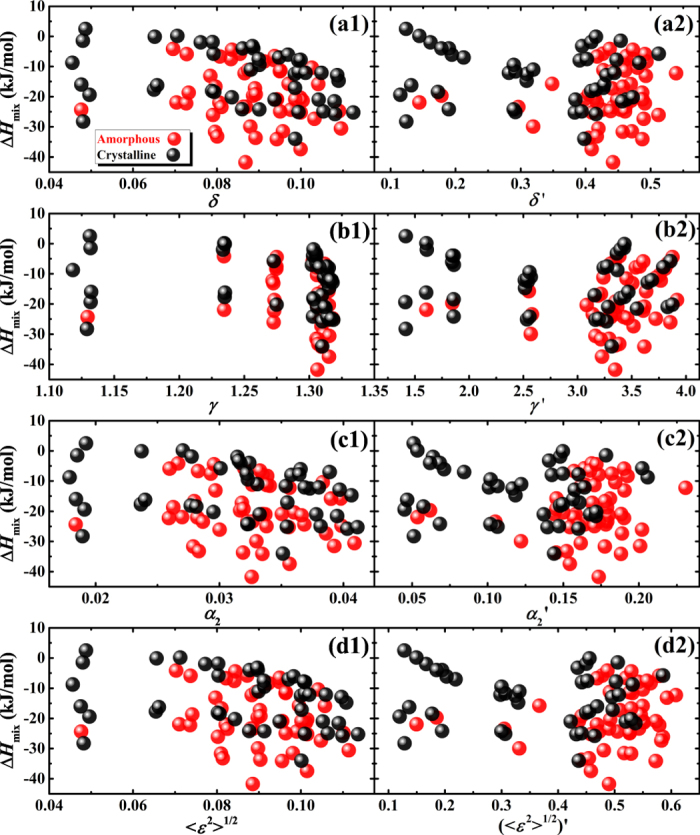
The plot of Δ*H*_mix_ vs. **(a1–d1)** original topological parameters and **(a2–d2)** modified topological parameters for all crystalline and amorphous alloys.

The explanations on the above mentioned four exceptional alloys are shown clearly in the plots of Δ*H*_mix_ vs. modified topological parameters in Fig. 7(a2–d2). More importantly, the regularity that amorphous alloys appearing in the lower right part of the plot of Δ*H*_mix_ vs. topological parameters can be observed in Fig. 7(a2–d2), which is supported by previous parametric approaches^5^,^31^,^32^,^33^,^34^,^35^,^36^,^37^,^38^. Furthermore, for all crystalline and amorphous alloys, the regularity shown in Fig. 8(a2–d2) is not as prominent as that shown in Fig. 7(a2–d2). However, it is still acceptable because most crystalline alloys in this work are intermetallic compounds, and even in HEAs with a wide compositional distribution^35^, it is much more difficult to distinguish amorphous alloys from intermetallic compounds than from solid solutions. By contrast, in Fig. 8(a1–d1), data points for amorphous alloys and crystalline alloys overlap to a large extent, and thus no regularity can be observed.

As discussed above, the effectiveness of topological parameters in separating the formation of amorphous and crystalline alloys is improved to a large extent through a simple modification of atomic radii. This improvement indicates that it is now necessary to reconsider whether the commonly used hard sphere assumption is still suitable to study the complex properties of alloys^55^. The modification proposed in this work, in spite of showing some improvement, is still based on existing parameters. New parameters, in which the electronic and topological factors are comprehensively taken into account at the very beginning, should be next target of further study on parametric approaches.

**Figure 9:**
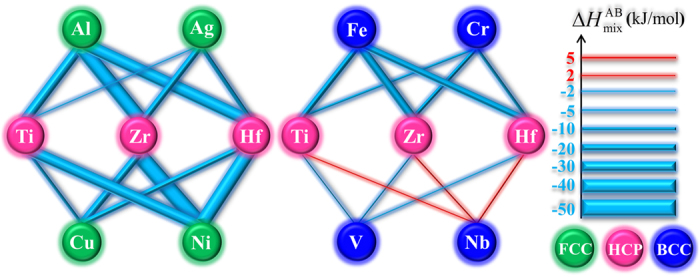
Mixing enthalpy between HCP (Ti, Zr, Hf) and FCC (Al, Ag, Cu, Ni) metals, HCP and BCC (Fe, V, Cr, Nb) metals.

**Figure 10:**
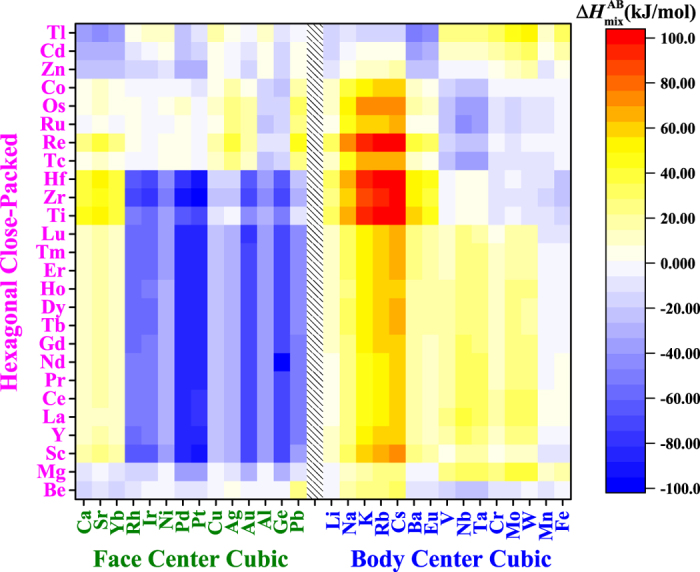
Mixing enthalpy of equiatomic binary liquid alloys composed of HCP and FCC metals, and HCP and BCC metals.

According to Fig. 4(a), Δ*H*_mix_ of most TiZrHf(F, FF, FFF) alloys are more negative than those of TiZrHf(B, BB, BBB) alloys. The cause of this observation is rationalized in Fig. 9. Clearly, compared to BCC metals, FCC metals have more negative Δ*H*_mix_ with HCP metals. This regularity actually not only holds in the 11 metals that are investigated in this work, but also in most metals, as shown in Fig. 10 that covers all solid-state metals^40^. There are in total 26 HCP metals, 14 FCC metals and 15 BCC metals. In 364 (26 × 14 = 364) equiatomic binary liquid alloys that are composed of HCP and FCC metals, 250 have negative Δ*H*_mix_. In 390 (26 × 15 = 390) alloys that are composed of HCP and BCC metals, 280 have positive Δ*H*_mix_. Indeed, a clear trend exists, but to the best of our knowledge^56^, this trend on how the combination of HCP and FCC or BCC elements can affect the mixing enthalpy has not been revealed before.

**Figure 11:**
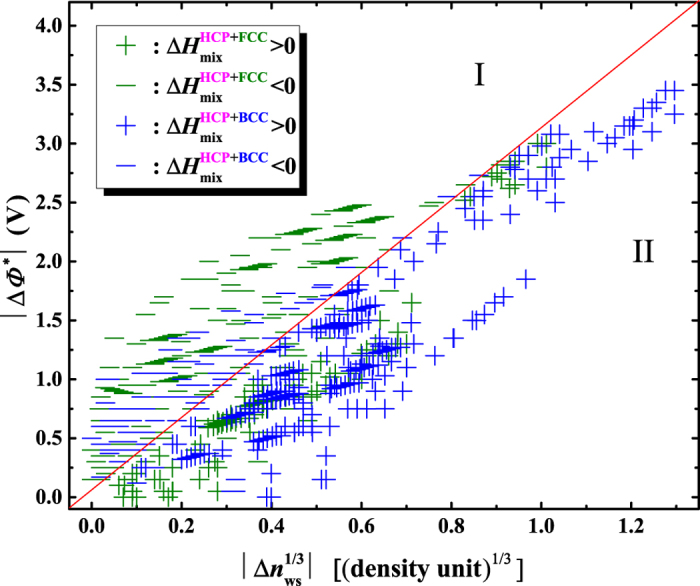
Signs of mixing enthalpy of equiatomic binary liquid alloys composed of HCP and FCC metals, and HCP and BCC metals. The signs are correlated with absolute values of 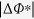 and 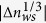, which are calculated using the data in ref. 58.

According to Miedema’s model^57^,^58^, the sign of 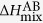 of binary AB alloys is mostly determined by the item 
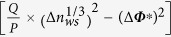
. Δ*n*_*ws*_ is the difference in electronic densities at the boundary of Wigner-Seitz cell of the pure metal A and B, which makes the electron densities be equal at the boundary in alloying. ΔΦ^*^ is the work function difference between the pure metal A and B, which determines the charge transfer in alloying. *Q*/*P* is a constant that is irrelevant to composition. Therefore, simply put, the sign of 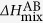 mostly depends on whether 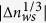 or 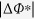 is relatively larger. In total 742 binary alloys that are composed of HCP and BCC, and HCP and FCC metals have non-zero mixing enthalpies. Figure 11 shows the distribution of these binary alloys in the 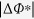 vs. 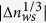 plot, where alloys are classified by their components and mixing enthalpy. The plot is divided into two regions separated by the diagonal. Mixing enthalpy are all negative in Region I because 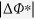 is relatively larger than 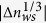. For the opposite reason, almost all mixing enthalpy are positive in Region II. In terms of alloying components, obviously most alloys composed of HCP and FCC metals distribute in Region I, while those composed of HCP and BCC metals distribute in Region II. Therefore, Fig. 11 points to such a scenario: most HCP and FCC metals have a relatively larger difference in work function and a smaller difference in cell boundary electronic density, therefore negative mixing enthalpy, and by contrast most HCP and BCC metals have relatively a smaller difference in work function and a larger difference in cell boundary electronic density, therefore positive mixing enthalpy. Such a scenario seems to indicate the existence of a hidden connection among several important concepts in the fields of metallurgy, crystallography and atomic physics. A deeper understanding for the hidden connection is beyond the main purpose of this work, and will be continued in the future studies.

Last but not least, what is covered in this study is basically to better distinguish the formation of the amorphous phase from crystalline phases, and it is not our intention here to predict the formation of bulk high-entropy amorphous alloys, or high-entropy bulk metallic glasses (HE-BMGs), although the latter is certainly an interesting and important topic. As a matter of fact, even predicting the compositions for conventional BMGs is not a well-solved problem, and predicting the compositions for HE-BMGs is certainly a more difficult one. It is hoped, however, that the new understanding obtained from this study, and future developments along the line of thinking, such as modification of conventionally used parameters (atomic radius in this case) and/or taking new perspectives into account (crystal structure of constituent elements in this case), can lead to the development guidelines for HE-BMGs, and enrich the current understanding on the glass forming ability in multi-component alloys.

## Conclusions

To summarize, the amorphous phase formation in 92 melt-spun equiatomic TiZrHfM, TiZrHfMM, TiZrHfMMM (M = Al, Ag, Cu, Ni, V, Cr, Nb, Fe) HEAs were systematically investigated by parametric approaches. Topological parameters using the atomic radii from pure elements were found to fail to distinguish the formation of amorphous alloys from crystalline alloys. Modified atomic radius is proposed by taking into account the change of the local electronic environment upon alloying. Its effectiveness is verified by a better agreement to experimentally measured lattice parameters using the Vegard’s law. In separating the formation of amorphous and crystalline HEAs, topological parameters utilizing modified atomic radius can work reasonably well. A combination of HCP and FCC metals was found to favor the amorphous phase formation, which was however not the case for a combination of HCP and BCC metals. Such an observation was rationalized by the fact that FCC metals have more negative mixing enthalpy with HCP metals than do BCC metals, which holds true in most metals. Findings from this work on the one hand provide parametric studies for high-entropy alloys with two perspectives, i.e., the modified atomic radius and the effect of crystalline structures of alloying elements, and on the other hand, possibly reveal a hidden connection among some important concepts in fields of metallurgy, crystallography and atomic physics.

## Method

The master alloys of TiZrHfM, TiZrHfMM, and TiZrHfMMM (where M = Al, Ag, Cu, Ni, V, Cr, Nb, Fe) alloys were prepared by arc melting the mixtures of metals with purities better than 99.95% in a Ti-gettered high-purity argon atmosphere. Ribbons with thickness of 15 ~ 20 μm and width of 1.0 ~ 1.5 mm were prepared via single roller melt spinning at a wheel surface velocity of 50 m/s (Rapid Quench Machine System VF-RQT50, Makabe Co. Ltd., Japan). The phase constitutions were determined by x-ray diffraction (XRD, Bruker D8) using the Cu-K*α* radiation and the working voltage and current of 40 kV and 200 mA, respectively. The parameters used in this work were calculated by a self-compiled program, which is now uploaded to the cloud and freely available to users^59^.

## Additional Information

**How to cite this article**: Hu, Q. *et al*. Parametric Study of Amorphous High-Entropy Alloys formation from two New Perspectives: Atomic Radius Modification and Crystalline Structure of Alloying Elements. *Sci. Rep.* **7**, 39917; doi: 10.1038/srep39917 (2017).

**Publisher's note:** Springer Nature remains neutral with regard to jurisdictional claims in published maps and institutional affiliations.

## Supplementary Material

Supplementary Information

## Figures and Tables

**Table 1:** Elemental characteristics^42^,^48^ of the metals used in this work.

	Ti	Zr	Hf	Fe	Cr	V	Nb	Al	Ag	Cu	Ni
ESN	4	5	6	4	4	4	5	3	5	4	4
VEC	4	4	4	8	6	5	5	3	11	11	10
ESN/VEC	1.000	1.250	1.500	0.500	0.667	0.800	1.000	1.000	0.455	0.364	0.400
*r* (Å)	1.462	1.603	1.578	1.241	1.249	1.316	1.429	1.432	1.445	1.278	1.246
Structure	HCP	HCP	HCP	BCC	BCC	BCC	BCC	FCC	FCC	FCC	FCC
