# Supplementary material for: Parametric Study of Amorphous High-Entropy Alloys formation from two New Perspectives: Atomic Radius Modification and Crystalline Structure of Alloying Elements
Source: Sci Rep. 2017 Jan 4;7:39917. doi: 10.1038/srep39917 (PMC5209706; doi:10.1038/srep39917)
Supplement: Supplementary Information [file srep39917-s1.doc]

**Supplementary Information**

**Parametric Study of Amorphous High-Entropy Alloys formation from Two New Perspectives: Atomic Radius Modification and Crystalline Structure of Alloying Elements**

**Q. Hu, S. Guo, J.M. Wang, Y.H. Yan, S.S. Chen, D.P. Lu, K.M. Liu, J.Z. Zou, X.R. Zeng**

Table S1. Comparison of the calculated and experimental lattice parameters of all BCC alloys (Pearson Symbol of cI2, Space Group of Im-3m(229)) constituted with the elements list in Table 1 and are available in PDF database of the version PDF-2 Release 2004. PDF-# is the PDF card number of the alloy. *r_i_*’/*r_i_* is the relative change in atomic radius of the *i*th element based on Eq.(8). *a* and *a*’ are lattice parameters calculated using Eq.(9) and (10), respectively. *a*_exp_ is the experimental lattice parameter given by the PDF card. *D* (*D*=(*a*-*a*_exp_)/ *a*_exp_) and *D*’ (*D*’=(*a*’-*a*_exp_)/ *a*_exp_) are the relative deviation of the calculated lattice parameters from experimental values. Signs of √ and × mean *a*’ are better and worse than *a* respectively; sign of = means *a*’ and *a* have similar or the same values.

| Alloys | PDF-# | *r*_1_’/*r*_1_  (%) | *r*_2_’/*r*_2_  (%) | *r*_3_’/*r*_3_  (%) | *a*  (Å) | *a*’  (Å) | *a*_exp_  (Å) | *D*  (‰) | *D*’  (‰) | Sign |
| --- | --- | --- | --- | --- | --- | --- | --- | --- | --- | --- |
| AlAg3 | 28-0033 | 50.0 | 110.0 |  | 3.33 | 3.166 | 3.25 | 24.5 | -25.7 | × |
| Al2V3 | 65-4730 | 85.7 | 107.1 |  | 3.146 | 3.088 | 3.069 | 25.2 | 6.1 | √ |
| AlNb7 | 65-5170 | 100.0 | 100.0 |  | 3.301 | 3.301 | 3.275 | 7.9 | 7.9 | = |
| Fe7Ni3 | 65-7251 | 93.0 | 116.3 |  | 2.869 | 2.87 | 2.861 | 2.9 | 3.1 | = |
| Cu3Fe17 | 65-7244 | 130.2 | 94.7 |  | 2.879 | 2.883 | 2.881 | -0.8 | 0.6 | = |
| V3Fe2 | 65-7053 | 80.6 | 129.0 |  | 2.97 | 2.95 | 2.92 | 17.1 | 10.2 | √ |
| AlV | 65-8509 | 87.5 | 109.4 |  | 3.173 | 3.109 | 3.074 | 32.2 | 11.3 | √ |
| NbTi | 65-9436 | 100.0 | 100.0 |  | 3.338 | 3.338 | 3.278 | 18.4 | 18.4 | = |
| TiNb | 65-9438 | 100.0 | 100.0 |  | 3.338 | 3.338 | 3.286 | 15.9 | 15.9 | = |
| NbV | 65-9439 | 90.0 | 112.5 |  | 3.17 | 3.195 | 3.183 | -4.2 | 3.6 | √ |
| ZrNb | 65-9440 | 88.9 | 111.1 |  | 3.501 | 3.479 | 3.439 | 18.0 | 11.6 | √ |
| Cr0.03Fe0.97 | 65-4607 | 75.6 | 100.8 |  | 2.867 | 2.866 | 2.869 | -0.9 | -0.9 | = |
| TiCr | 65-9021 | 80.0 | 120.0 |  | 3.13 | 3.081 | 3.12 | 3.3 | -12.4 | × |
| CrFe4 | 65-4664 | 78.9 | 105.3 |  | 2.87 | 2.869 | 2.866 | 1.3 | 1.0 | = |
| Fe0.975Ti0.025 | 65-9141 | 101.3 | 50.6 |  | 2.879 | 2.872 | 2.872 | 2.3 | 0.1 | √ |
| V3Fe7 | 65-5132 | 70.4 | 112.7 |  | 2.918 | 2.903 | 2.89 | 9.7 | 4.3 | √ |
| TiV | 65-7658 | 88.9 | 111.1 |  | 3.208 | 3.189 | 3.14 | 21.6 | 15.6 | √ |
| AlV3 | 65-5142 | 87.5 | 109.4 |  | 3.173 | 3.109 | 3.058 | 37.6 | 16.6 | √ |
| TiV | 65-4979 | 88.9 | 111.1 |  | 3.208 | 3.189 | 3.143 | 20.6 | 14.6 | √ |
| Fe9.64Ti0.36 | 65-7743 | 101.8 | 50.9 |  | 2.884 | 2.875 | 2.877 | 2.6 | -0.6 | √ |
| Fe19Ni | 65-7752 | 98.8 | 123.5 |  | 2.867 | 2.867 | 2.868 | -0.5 | -0.5 | = |
| Fe10.8Ni | 65-7753 | 97.9 | 122.4 |  | 2.867 | 2.867 | 2.871 | -1.4 | -1.3 | = |
| Cr1.07Fe18.93 | 65-7775 | 76.0 | 101.4 |  | 2.867 | 2.867 | 2.869 | -0.7 | -0.8 | = |
| Ag0.7Fe0.3 | 65-8447 | 102.4 | 93.1 |  | 3.196 | 3.192 | 2.96 | 79.6 | 78.3 | = |
| Fe4V | 65-6842 | 108.1 | 67.6 |  | 2.901 | 2.889 | 2.886 | 5.1 | 1.2 | √ |
| VCr | 65-6819 | 90.9 | 109.1 |  | 2.962 | 2.955 | 2.95 | 4.0 | 1.6 | √ |
| CrTi4 | 65-6818 | 136.4 | 90.9 |  | 3.278 | 3.242 | 3.21 | 21.2 | 10.0 | √ |
| Al3Cr7 | 65-6108 | 72.5 | 108.8 |  | 3.011 | 2.917 | 2.96 | 17.3 | -14.5 | √ |
| Fe9V | 65-7509 | 103.9 | 64.9 |  | 2.883 | 2.877 | 2.878 | 1.8 | -0.3 | √ |
| V2Cr2Fe | 65-6315 | 83.3 | 100.0 | 133.3 | 2.943 | 2.931 | 2.9 | 14.7 | 10.7 | √ |
| VCrFe8 | 65-6033 | 66.7 | 80.0 | 106.7 | 2.885 | 2.879 | 2.88 | 1.8 | -0.4 | √ |
| Al0.12Nb0.38Ti0.5 | 53-0483 | 100.0 | 100.0 | 100.0 | 3.339 | 3.339 | 3.244 | 29.3 | 29.3 | = |
| Fe2AlCr | 54-0387 | 120.0 | 60.0 | 90.0 | 2.981 | 2.865 | 2.894 | 30.0 | -10.1 | √ |
| Fe0.1Ti0.18V0.72 | 65-9142 | 156.3 | 78.1 | 97.7 | 3.083 | 3.06 | 3.04 | 14.0 | 6.4 | √ |
| AlNbV2 | 65-5883 | 88.9 | 88.9 | 111.1 | 3.171 | 3.157 | 3.123 | 15.5 | 10.8 | √ |
| AlFeV2 | 65-4460 | 71.4 | 142.9 | 89.3 | 3.063 | 2.971 | 2.97 | 31.3 | 0.3 | √ |
| VCrFe8 | 65-6033 | 66.7 | 80.0 | 106.7 | 2.885 | 2.879 | 2.88 | 1.8 | -0.4 | √ |
| V2Cr2Fe | 65-6315 | 83.3 | 100.0 | 133.3 | 2.943 | 2.931 | 2.9 | 14.7 | 10.7 | √ |
| HfZrNb3 | 65-7471 | 75.4 | 90.4 | 113.0 | 3.449 | 3.457 | 3.4 | 14.5 | 16.8 | × |

Table S2. Modified atomic radius *r_i_*' of the *i*th element in HEAs.

| Categories | Alloys* | *r*_1_' (Å) | *r*_2_' (Å) | *r*_3_' (Å) | *r*_4_' (Å) | *r*_5_' (Å) | *r*_6_' (Å) | Phase* |
| --- | --- | --- | --- | --- | --- | --- | --- | --- |
| TiZrHfB | Fe | 1.388 | 1.218 | 0.999 | 2.358 |  |  | Am |
|  | Cr | 1.543 | 1.353 | 1.110 | 1.978 |  |  | SS+IC |
|  | V | 1.633 | 1.433 | 1.175 | 1.839 |  |  | SS+IC |
|  | Nb | 1.719 | 1.508 | 1.237 | 1.681 |  |  | IC |
| TiZrHfF | Al | 1.754 | 1.538 | 1.262 | 1.718 |  |  | IC |
|  | Ag | 1.271 | 1.115 | 0.914 | 2.764 |  |  | IC |
|  | Cu | 1.207 | 1.059 | 0.869 | 2.903 |  |  | Am |
|  | Ni | 1.262 | 1.107 | 0.908 | 2.690 |  |  | Am |
| TiZrHfBB | FeCr | 1.293 | 1.134 | 0.930 | 2.196 | 1.657 |  | IC |
|  | FeV | 1.345 | 1.179 | 0.968 | 2.284 | 1.513 |  | IC |
|  | FeNb | 1.403 | 1.231 | 1.010 | 2.383 | 1.372 |  | IC |
|  | CrV | 1.462 | 1.282 | 1.052 | 1.874 | 1.645 |  | IC |
|  | CrNb | 1.525 | 1.338 | 1.097 | 1.955 | 1.491 |  | IC |
|  | VNb | 1.594 | 1.399 | 1.147 | 1.795 | 1.559 |  | IC |
| TiZrHfFF | AlAg | 1.293 | 1.134 | 0.930 | 1.267 | 2.812 |  | Am |
|  | AlCu | 1.237 | 1.085 | 0.890 | 1.211 | 2.974 |  | Am |
|  | AlNi | 1.286 | 1.128 | 0.925 | 1.260 | 2.741 |  | Am |
|  | AgCu | 1.032 | 0.905 | 0.742 | 2.244 | 2.481 |  | Am |
|  | AgNi | 1.063 | 0.932 | 0.765 | 2.312 | 2.265 |  | Am |
|  | CuNi | 1.019 | 0.894 | 0.733 | 2.450 | 2.171 |  | Am |
| TiZrHfBBB | FeCrV | 1.273 | 1.117 | 0.916 | 2.162 | 1.632 | 1.433 | IC |
|  | FeCrNb | 1.320 | 1.158 | 0.950 | 2.242 | 1.692 | 1.291 | IC |
|  | FeVNb | 1.364 | 1.197 | 0.982 | 2.317 | 1.535 | 1.334 | IC |
|  | CrVNb | 1.461 | 1.282 | 1.052 | 1.874 | 1.645 | 1.429 | IC |
| TiZrHfFFF | AlAgCu | 1.066 | 0.936 | 0.767 | 1.045 | 2.319 | 2.565 | Am |
|  | AlAgNi | 1.096 | 0.961 | 0.789 | 1.074 | 2.384 | 2.336 | Am |
|  | AlCuNi | 1.056 | 0.926 | 0.760 | 1.034 | 2.538 | 2.250 | Am |
|  | AgCuNi | 0.930 | 0.816 | 0.669 | 2.023 | 2.237 | 1.982 | Am |
| TiZrHfBF | FeAl | 1.398 | 1.226 | 1.006 | 2.374 | 1.369 |  | Am |
|  | FeAg | 1.131 | 0.993 | 0.814 | 1.922 | 2.461 |  | Am |
|  | FeCu | 1.084 | 0.951 | 0.780 | 1.842 | 2.608 |  | Am |
|  | FeNi | 1.120 | 0.983 | 0.806 | 1.903 | 2.388 |  | Am |
|  | CrAl | 1.531 | 1.343 | 1.102 | 1.963 | 1.500 |  | IC |
|  | CrAg | 1.210 | 1.061 | 0.870 | 1.551 | 2.630 |  | Am |
|  | CrCu | 1.159 | 1.017 | 0.834 | 1.486 | 2.787 |  | Am+Cry |
|  | CrNi | 1.201 | 1.053 | 0.864 | 1.539 | 2.559 |  | IC |
|  | VAl | 1.608 | 1.410 | 1.157 | 1.809 | 1.575 |  | Am |
|  | VAg | 1.253 | 1.099 | 0.901 | 1.410 | 2.724 |  | Am |
|  | VCu | 1.201 | 1.053 | 0.864 | 1.351 | 2.887 |  | Am |
|  | VNi | 1.245 | 1.092 | 0.896 | 1.401 | 2.653 |  | Am |
|  | NbAl | 1.681 | 1.474 | 1.209 | 1.643 | 1.646 |  | IC |
|  | NbAg | 1.305 | 1.145 | 0.939 | 1.276 | 2.838 |  | IC |
|  | NbCu | 1.253 | 1.099 | 0.901 | 1.225 | 3.012 |  | SS |
|  | NbNi | 1.299 | 1.140 | 0.935 | 1.270 | 2.769 |  | Am |
| TiZrHfBFF | FeAlAg | 1.161 | 1.018 | 0.835 | 1.971 | 1.137 | 2.524 | Am |
|  | FeAlCu | 1.118 | 0.980 | 0.804 | 1.898 | 1.095 | 2.688 | Am |
|  | FeAlNi | 1.151 | 1.010 | 0.829 | 1.956 | 1.128 | 2.454 | Am |
|  | FeAgCu | 0.974 | 0.855 | 0.701 | 1.655 | 2.119 | 2.343 | Am |
|  | FeAgNi | 0.998 | 0.876 | 0.718 | 1.695 | 2.171 | 2.127 | Am |
|  | FeCuNi | 0.962 | 0.844 | 0.693 | 1.635 | 2.314 | 2.051 | Am |
|  | CrAlAg | 1.233 | 1.082 | 0.887 | 1.581 | 1.208 | 2.682 | IC |
|  | CrAlCu | 1.187 | 1.042 | 0.854 | 1.522 | 1.163 | 2.856 | IC |
|  | CrAlNi | 1.226 | 1.075 | 0.882 | 1.571 | 1.201 | 2.612 | IC |
|  | CrAgCu | 1.023 | 0.897 | 0.736 | 1.312 | 2.225 | 2.460 | Am |
|  | CrAgNi | 1.049 | 0.920 | 0.755 | 1.345 | 2.282 | 2.236 | IC |
|  | CrCuNi | 1.012 | 0.888 | 0.728 | 1.297 | 2.433 | 2.156 | IC |
|  | VAlAg | 1.273 | 1.117 | 0.916 | 1.433 | 1.247 | 2.768 | IC |
|  | VAlCu | 1.226 | 1.075 | 0.882 | 1.380 | 1.201 | 2.948 | IC |
|  | VAlNi | 1.267 | 1.111 | 0.911 | 1.426 | 1.241 | 2.699 | Am |
|  | VAgCu | 1.049 | 0.920 | 0.755 | 1.181 | 2.282 | 2.523 | Am |
|  | VAgNi | 1.077 | 0.945 | 0.775 | 1.212 | 2.342 | 2.295 | Am |
|  | VCuNi | 1.038 | 0.911 | 0.747 | 1.169 | 2.497 | 2.213 | Am |
|  | NbAlAg | 1.320 | 1.158 | 0.950 | 1.291 | 1.293 | 2.871 | IC |
|  | NbAlCu | 1.273 | 1.117 | 0.916 | 1.245 | 1.247 | 3.061 | Am |
|  | NbAlNi | 1.315 | 1.154 | 0.946 | 1.286 | 1.289 | 2.803 | Am |
|  | NbAgCu | 1.087 | 0.953 | 0.782 | 1.063 | 2.363 | 2.613 | Am |
|  | NbAgNi | 1.115 | 0.978 | 0.803 | 1.091 | 2.426 | 2.377 | Am+Cry |
|  | NbCuNi | 1.077 | 0.945 | 0.775 | 1.053 | 2.590 | 2.295 | Am |
| TiZrHfBBF | FeCrAl | 1.310 | 1.149 | 0.943 | 2.226 | 1.680 | 1.284 | IC |
|  | FeCrAg | 1.106 | 0.970 | 0.796 | 1.879 | 1.418 | 2.405 | IC |
|  | FeCrCu | 1.066 | 0.936 | 0.767 | 1.811 | 1.367 | 2.565 | IC |
|  | FeCrNi | 1.096 | 0.961 | 0.789 | 1.862 | 1.405 | 2.336 | IC |
|  | FeVAl | 1.357 | 1.190 | 0.977 | 2.305 | 1.527 | 1.329 | IC |
|  | FeVAg | 1.137 | 0.997 | 0.818 | 1.931 | 1.279 | 2.472 | IC |
|  | FeVCu | 1.096 | 0.961 | 0.789 | 1.862 | 1.234 | 2.636 | IC |
|  | FeVNi | 1.127 | 0.989 | 0.811 | 1.915 | 1.269 | 2.403 | IC |
|  | FeNbAl | 1.409 | 1.236 | 1.014 | 2.394 | 1.378 | 1.381 | Am |
|  | FeNbAg | 1.177 | 1.033 | 0.847 | 2.000 | 1.151 | 2.560 | Am |
|  | FeNbCu | 1.137 | 0.997 | 0.818 | 1.931 | 1.111 | 2.733 | Am |
|  | FeNbNi | 1.169 | 1.026 | 0.841 | 1.986 | 1.143 | 2.492 | Am |
|  | CrVAl | 1.462 | 1.282 | 1.052 | 1.874 | 1.645 | 1.432 | Am |
|  | CrVAg | 1.204 | 1.056 | 0.866 | 1.543 | 1.355 | 2.617 | IC |
|  | CrVCu | 1.161 | 1.018 | 0.835 | 1.488 | 1.306 | 2.791 | IC |
|  | CrVNi | 1.196 | 1.049 | 0.860 | 1.533 | 1.346 | 2.548 | IC |
|  | CrNbAl | 1.518 | 1.331 | 1.092 | 1.946 | 1.484 | 1.487 | IC |
|  | CrNbAg | 1.247 | 1.093 | 0.897 | 1.598 | 1.219 | 2.711 | IC |
|  | CrNbCu | 1.204 | 1.056 | 0.866 | 1.543 | 1.177 | 2.894 | Am |
|  | CrNbNi | 1.240 | 1.088 | 0.892 | 1.590 | 1.212 | 2.643 | Am |
|  | VNbAl | 1.578 | 1.385 | 1.136 | 1.777 | 1.543 | 1.546 | IC |
|  | VNbAg | 1.284 | 1.127 | 0.924 | 1.446 | 1.256 | 2.793 | IC |
|  | VNbCu | 1.240 | 1.088 | 0.892 | 1.396 | 1.212 | 2.982 | Am |
|  | VNbNi | 1.279 | 1.122 | 0.920 | 1.439 | 1.250 | 2.725 | Am |

Table S3. List of chemical parameters, original and modified topological parameters, and phase constitutions of HEAs. The unit of Δ*H*_mix_ is kJ/mol.

| Cate-  gories* | Alloys* | Δ*H*_mix_ | *δ* | *γ* | *α*_2_ | <*ε* ^2^>^1/2^ | *δ *' | *γ *' | *α*_2_' | (<*ε* ^2^>^1/2^)' | Phase* |
| --- | --- | --- | --- | --- | --- | --- | --- | --- | --- | --- | --- |
| TiZrHfB | Fe | -15.75 | 0.097 | 1.311 | 0.04 | 0.1 | 0.348 | 2.55 | 0.145 | 0.367 | Am |
|  | Cr | -7 | 0.095 | 1.302 | 0.039 | 0.097 | 0.212 | 1.858 | 0.084 | 0.219 | SS+IC |
|  | V | -2 | 0.076 | 1.234 | 0.031 | 0.077 | 0.161 | 1.609 | 0.064 | 0.167 | SS+IC |
|  | Nb | 2.5 | 0.049 | 1.131 | 0.019 | 0.049 | 0.124 | 1.415 | 0.051 | 0.128 | IC |
| TiZrHfF | Al | -28.25 | 0.048 | 1.129 | 0.019 | 0.048 | 0.124 | 1.415 | 0.051 | 0.128 | IC |
|  | Ag | -8.75 | 0.046 | 1.118 | 0.018 | 0.046 | 0.482 | 3.368 | 0.206 | 0.532 | IC |
|  | Cu | -12.25 | 0.087 | 1.272 | 0.036 | 0.088 | 0.539 | 3.767 | 0.231 | 0.608 | Am |
|  | Ni | -31.5 | 0.096 | 1.306 | 0.039 | 0.098 | 0.471 | 3.293 | 0.201 | 0.517 | Am |
| TiZrHfBB | FeCr | -14.72 | 0.109 | 1.315 | 0.041 | 0.111 | 0.309 | 2.514 | 0.118 | 0.329 | IC |
|  | FeV | -12.48 | 0.099 | 1.314 | 0.037 | 0.1 | 0.309 | 2.541 | 0.116 | 0.324 | IC |
|  | FeNb | -11.04 | 0.088 | 1.311 | 0.033 | 0.09 | 0.319 | 2.568 | 0.122 | 0.332 | IC |
|  | CrV | -6.08 | 0.097 | 1.305 | 0.037 | 0.098 | 0.194 | 1.844 | 0.071 | 0.203 | IC |
|  | CrNb | -4 | 0.086 | 1.303 | 0.032 | 0.088 | 0.19 | 1.858 | 0.068 | 0.195 | IC |
|  | VNb | 0.16 | 0.071 | 1.235 | 0.027 | 0.071 | 0.144 | 1.605 | 0.053 | 0.149 | IC |
| TiZrHfFF | AlAg | -24.32 | 0.048 | 1.13 | 0.018 | 0.048 | 0.454 | 3.427 | 0.178 | 0.505 | Am |
|  | AlCu | -26.08 | 0.079 | 1.273 | 0.03 | 0.08 | 0.512 | 3.854 | 0.202 | 0.585 | Am |
|  | AlNi | -41.76 | 0.087 | 1.306 | 0.033 | 0.089 | 0.442 | 3.347 | 0.173 | 0.49 | Am |
|  | AgCu | -13.12 | 0.078 | 1.272 | 0.03 | 0.08 | 0.493 | 3.506 | 0.191 | 0.592 | Am |
|  | AgNi | -23.36 | 0.086 | 1.306 | 0.032 | 0.088 | 0.461 | 3.14 | 0.179 | 0.543 | Am |
|  | CuNi | -27.36 | 0.103 | 1.309 | 0.039 | 0.105 | 0.489 | 3.517 | 0.189 | 0.583 | Am |
| TiZrHfBBB | FeCrV | -12.111 | 0.105 | 1.317 | 0.038 | 0.106 | 0.282 | 2.513 | 0.101 | 0.298 | IC |
|  | FeCrNb | -11.667 | 0.099 | 1.315 | 0.035 | 0.101 | 0.292 | 2.534 | 0.106 | 0.306 | IC |
|  | FeVNb | -9.444 | 0.09 | 1.314 | 0.032 | 0.091 | 0.289 | 2.557 | 0.102 | 0.3 | IC |
|  | CrVNb | -4 | 0.089 | 1.305 | 0.032 | 0.09 | 0.178 | 1.845 | 0.062 | 0.185 | IC |
| TiZrHfFFF | AlAgCu | -22.222 | 0.073 | 1.273 | 0.026 | 0.074 | 0.491 | 3.607 | 0.19 | 0.579 | Am |
|  | AlAgNi | -31.667 | 0.08 | 1.307 | 0.028 | 0.081 | 0.457 | 3.216 | 0.177 | 0.529 | Am |
|  | AlCuNi | -34.111 | 0.094 | 1.309 | 0.033 | 0.096 | 0.487 | 3.617 | 0.188 | 0.573 | Am |
|  | AgCuNi | -21 | 0.094 | 1.309 | 0.034 | 0.096 | 0.448 | 3.367 | 0.158 | 0.551 | Am |
| TiZrHfBF | FeAl | -29.92 | 0.088 | 1.311 | 0.033 | 0.09 | 0.319 | 2.567 | 0.122 | 0.331 | Am |
|  | FeAg | -11.2 | 0.087 | 1.311 | 0.033 | 0.089 | 0.427 | 3.239 | 0.165 | 0.492 | Am |
|  | FeCu | -15.84 | 0.104 | 1.315 | 0.039 | 0.106 | 0.469 | 3.633 | 0.181 | 0.55 | Am |
|  | FeNi | -30.56 | 0.11 | 1.315 | 0.041 | 0.111 | 0.42 | 3.165 | 0.161 | 0.481 | Am |
|  | CrAl | -24.16 | 0.086 | 1.303 | 0.032 | 0.088 | 0.19 | 1.858 | 0.068 | 0.195 | IC |
|  | CrAg | -5.76 | 0.085 | 1.303 | 0.032 | 0.087 | 0.426 | 3.344 | 0.164 | 0.478 | Am |
|  | CrCu | -10.4 | 0.103 | 1.306 | 0.038 | 0.104 | 0.48 | 3.757 | 0.184 | 0.55 | Am+Cry |
|  | CrNi | -25.76 | 0.108 | 1.31 | 0.04 | 0.11 | 0.416 | 3.267 | 0.16 | 0.465 | IC |
|  | VAl | -21.92 | 0.07 | 1.235 | 0.027 | 0.071 | 0.144 | 1.605 | 0.054 | 0.15 | Am |
|  | VAg | -4.16 | 0.07 | 1.234 | 0.027 | 0.07 | 0.437 | 3.386 | 0.169 | 0.487 | Am |
|  | VCu | -8.32 | 0.091 | 1.275 | 0.034 | 0.092 | 0.494 | 3.806 | 0.192 | 0.564 | Am |
|  | VNi | -24.32 | 0.098 | 1.309 | 0.037 | 0.099 | 0.426 | 3.307 | 0.164 | 0.473 | Am |
|  | NbAl | -19.36 | 0.05 | 1.132 | 0.019 | 0.05 | 0.115 | 1.411 | 0.045 | 0.12 | IC |
|  | NbAg | -1.44 | 0.048 | 1.132 | 0.019 | 0.048 | 0.454 | 3.428 | 0.178 | 0.506 | IC |
|  | NbCu | -5.76 | 0.079 | 1.273 | 0.03 | 0.08 | 0.512 | 3.855 | 0.202 | 0.586 | SS |
|  | NbNi | -23.36 | 0.087 | 1.306 | 0.033 | 0.089 | 0.442 | 3.347 | 0.174 | 0.49 | Am |
| TiZrHfBFF | FeAlAg | -22 | 0.081 | 1.312 | 0.028 | 0.082 | 0.417 | 3.304 | 0.157 | 0.469 | Am |
|  | FeAlCu | -24.889 | 0.095 | 1.315 | 0.034 | 0.096 | 0.461 | 3.721 | 0.171 | 0.526 | Am |
|  | FeAlNi | -37.444 | 0.1 | 1.315 | 0.036 | 0.101 | 0.409 | 3.226 | 0.154 | 0.458 | Am |
|  | FeAgCu | -11.556 | 0.095 | 1.314 | 0.034 | 0.096 | 0.441 | 3.452 | 0.162 | 0.532 | Am |
|  | FeAgNi | -20.333 | 0.1 | 1.315 | 0.036 | 0.102 | 0.414 | 3.084 | 0.15 | 0.492 | Am |
|  | FeCuNi | -24.778 | 0.109 | 1.318 | 0.04 | 0.11 | 0.438 | 3.46 | 0.161 | 0.527 | Am |
|  | CrAlAg | -18.111 | 0.079 | 1.303 | 0.028 | 0.08 | 0.409 | 3.396 | 0.148 | 0.45 | IC |
|  | CrAlCu | -21 | 0.094 | 1.306 | 0.033 | 0.095 | 0.463 | 3.831 | 0.168 | 0.52 | IC |
|  | CrAlNi | -34 | 0.099 | 1.31 | 0.035 | 0.1 | 0.398 | 3.315 | 0.144 | 0.436 | IC |
|  | CrAgCu | -7.889 | 0.094 | 1.306 | 0.033 | 0.095 | 0.46 | 3.539 | 0.173 | 0.542 | Am |
|  | CrAgNi | -17.111 | 0.099 | 1.31 | 0.035 | 0.1 | 0.427 | 3.158 | 0.161 | 0.497 | IC |
|  | CrCuNi | -21.556 | 0.108 | 1.313 | 0.04 | 0.109 | 0.456 | 3.548 | 0.171 | 0.537 | IC |
|  | VAlAg | -17.667 | 0.065 | 1.235 | 0.024 | 0.065 | 0.416 | 3.433 | 0.15 | 0.455 | IC |
|  | VAlCu | -20.222 | 0.084 | 1.275 | 0.03 | 0.084 | 0.472 | 3.874 | 0.172 | 0.529 | IC |
|  | VAlNi | -33.667 | 0.089 | 1.309 | 0.032 | 0.09 | 0.405 | 3.351 | 0.145 | 0.441 | Am |
|  | VAgCu | -7.556 | 0.083 | 1.275 | 0.029 | 0.084 | 0.474 | 3.573 | 0.182 | 0.557 | Am |
|  | VAgNi | -17.222 | 0.089 | 1.309 | 0.032 | 0.09 | 0.44 | 3.188 | 0.169 | 0.509 | Am |
|  | VCuNi | -21.333 | 0.1 | 1.311 | 0.037 | 0.101 | 0.47 | 3.583 | 0.18 | 0.552 | Am |
|  | NbAlAg | -16 | 0.048 | 1.132 | 0.018 | 0.048 | 0.429 | 3.469 | 0.157 | 0.469 | IC |
|  | NbAlCu | -18.667 | 0.073 | 1.273 | 0.026 | 0.074 | 0.487 | 3.917 | 0.179 | 0.546 | Am |
|  | NbAlNi | -33.222 | 0.08 | 1.307 | 0.028 | 0.081 | 0.417 | 3.385 | 0.152 | 0.454 | Am |
|  | NbAgCu | -5.889 | 0.073 | 1.273 | 0.026 | 0.074 | 0.491 | 3.607 | 0.19 | 0.579 | Am |
|  | NbAgNi | -16.667 | 0.08 | 1.307 | 0.028 | 0.081 | 0.457 | 3.216 | 0.178 | 0.529 | Am+Cry |
|  | NbCuNi | -20.889 | 0.094 | 1.31 | 0.033 | 0.096 | 0.487 | 3.617 | 0.188 | 0.573 | Am |
| TiZrHfBBF | FeCrAl | -25.111 | 0.099 | 1.315 | 0.035 | 0.101 | 0.292 | 2.534 | 0.106 | 0.306 | IC |
|  | FeCrAg | -8 | 0.099 | 1.315 | 0.036 | 0.101 | 0.391 | 3.242 | 0.142 | 0.442 | IC |
|  | FeCrCu | -12.889 | 0.109 | 1.318 | 0.04 | 0.11 | 0.432 | 3.647 | 0.157 | 0.495 | IC |
|  | FeCrNi | -25.222 | 0.112 | 1.318 | 0.041 | 0.114 | 0.383 | 3.166 | 0.14 | 0.432 | IC |
|  | FeVAl | -24.222 | 0.09 | 1.314 | 0.032 | 0.091 | 0.289 | 2.557 | 0.102 | 0.3 | IC |
|  | FeVAg | -7.556 | 0.09 | 1.314 | 0.032 | 0.091 | 0.402 | 3.273 | 0.15 | 0.452 | IC |
|  | FeVCu | -12.111 | 0.101 | 1.317 | 0.037 | 0.102 | 0.444 | 3.685 | 0.164 | 0.508 | IC |
|  | FeVNi | -24.889 | 0.105 | 1.317 | 0.038 | 0.106 | 0.394 | 3.197 | 0.147 | 0.441 | IC |
|  | FeNbAl | -23.444 | 0.081 | 1.312 | 0.029 | 0.082 | 0.296 | 2.579 | 0.105 | 0.305 | Am |
|  | FeNbAg | -6.667 | 0.081 | 1.312 | 0.028 | 0.082 | 0.418 | 3.304 | 0.157 | 0.469 | Am |
|  | FeNbCu | -11.333 | 0.095 | 1.315 | 0.034 | 0.096 | 0.461 | 3.722 | 0.171 | 0.527 | Am |
|  | FeNbNi | -25.222 | 0.1 | 1.315 | 0.036 | 0.102 | 0.41 | 3.226 | 0.154 | 0.458 | Am |
|  | CrVAl | -19.667 | 0.089 | 1.305 | 0.032 | 0.09 | 0.178 | 1.845 | 0.062 | 0.185 | Am |
|  | CrVAg | -3.222 | 0.088 | 1.305 | 0.032 | 0.09 | 0.395 | 3.363 | 0.141 | 0.435 | IC |
|  | CrVCu | -7.778 | 0.1 | 1.308 | 0.036 | 0.101 | 0.447 | 3.791 | 0.16 | 0.502 | IC |
|  | CrVNi | -21 | 0.104 | 1.312 | 0.038 | 0.105 | 0.384 | 3.283 | 0.137 | 0.422 | IC |
|  | CrNbAl | -18.444 | 0.079 | 1.304 | 0.028 | 0.081 | 0.173 | 1.857 | 0.057 | 0.177 | IC |
|  | CrNbAg | -1.889 | 0.079 | 1.303 | 0.028 | 0.08 | 0.409 | 3.397 | 0.148 | 0.45 | IC |
|  | CrNbCu | -6.556 | 0.094 | 1.306 | 0.033 | 0.095 | 0.463 | 3.831 | 0.168 | 0.52 | Am |
|  | CrNbNi | -20.889 | 0.099 | 1.31 | 0.035 | 0.1 | 0.398 | 3.316 | 0.144 | 0.437 | Am |
|  | VNbAl | -16.222 | 0.066 | 1.235 | 0.024 | 0.066 | 0.132 | 1.603 | 0.047 | 0.137 | IC |
|  | VNbAg | -0.111 | 0.065 | 1.235 | 0.024 | 0.066 | 0.416 | 3.433 | 0.15 | 0.455 | IC |
|  | VNbCu | -4.444 | 0.084 | 1.275 | 0.03 | 0.084 | 0.473 | 3.875 | 0.172 | 0.53 | Am |
|  | VNbNi | -19.222 | 0.089 | 1.309 | 0.032 | 0.09 | 0.405 | 3.351 | 0.145 | 0.441 | Am |

*Notes in Table S2 and S3:

-B and F in the row of Categories are short for BCC and FCC metals, respectively.

-The first three elements “TiZrHf” are omitted in the row of Alloys.

-Am, Cry, IC, SS in the row of Phase are short for amorphous, crystalline, intermetallic compound, and solid solution, respectively.
